# A systematic review and meta-analysis of weight loss in control group participants of lifestyle randomized trials

**DOI:** 10.1038/s41598-022-15770-x

**Published:** 2022-07-18

**Authors:** Amira Bouzalmate Hajjaj, Paloma Massó Guijarro, Khalid Saeed Khan, Aurora Bueno-Cavanillas, Naomi Cano-Ibáñez

**Affiliations:** 1 Department of Preventive Medicine and Public Health, Faculty of Medicine, University of Granada, Campus de la Salud. Avda. de la Investigación 11, 18016 Granada, Spain; 2 Preventive Medicine Unit, Universitary Hospital Virgen de Las Nieves, Granada, Spain; 3 CIBER de Epidemiología Y Salud Pública (CIBERESP-Spain), Madrid, Spain; 4 Instituto de Investigación Biosanitaria de Granada (IBS.GRANADA), Granada, Spain

**Keywords:** Outcomes research, Lifestyle modification, Weight management, Randomized controlled trials, Obesity, Epidemiology

## Abstract

Randomized clinical trials (RCTs) of lifestyle modification have reported beneficial effects of interventions, compared to control. Whether participation in the control group has benefits is unknown. To determine whether control group participants experience weight loss during the course of RCTs. After prospective registration (PROSPERO CRD42021233070), we conducted searches in Medline, Scopus, Web of Science, Cochrane library and Clinicaltrials.gov databases from inception to May 2021 without language restriction to capture RCTs on dietary advice or physical activity interventions in adults with overweight, obesity or metabolic syndrome. Data extraction and study quality assessment was performed by two independent reviewers. Weight loss in the control group, i.e., the difference between baseline and post-intervention, was pooled using random effects model generating mean difference and 95% confidence interval (CI). Heterogeneity was assessed using the I^2^ statistical test. Subgroup meta-analysis was performed stratifying by follow-up period, type of control group protocols and high-quality studies. Among the 22 included studies (4032 participants), the risk of bias was low in 9 (40%) studies. Overall, the controls groups experienced weight loss of − 0.41 kg (95% CI − 0.53 to − 0.28; I^2^ = 73.5% *p* < 0.001). To identify a result that is an outlier, we inspected the forest plot for spread of the point estimates and the confidence intervals. The magnitude of the benefit was related to the duration of follow-up (− 0.51 kg, 95% CI − 0.68, − 0.3, for 1–4 months follow-up; − 0.32 kg, 95% CI − 0.58, − 0.07, 5–12 months; − 0.20 kg, 95% CI − 0.49, 0.10, ≥ 12 months). In high-quality studies we found an overall weight loss mean difference of − 0.16 (95% CI − 0.39, 0.09) with a considerable heterogeneity (I^2^ = 74%; *p* < 0.000). Among studies including control group in waiting lists and combining standard care, advice and material, no heterogeneity was found (I^2^ = 0%, *p* = 0.589) and (I^2^ = 0%, *p* = 0.438); and the mean difference was − 0.84 kg (95% CI − 2.47, 0.80) and − 0.65 kg (95% CI − 1.03, − 0.27) respectively. Participation in control groups of RCTs of lifestyle interventions had a benefit in terms of weight loss in meta-analysis with heterogeneity. These results should be used to interpret the benefits observed with respect to intervention effect in trials. That control groups accrue benefits should be included in patient information sheets to encourage participation in future trials among patients with overweight and obesity.

## Introduction

Obesity, a major cause of morbidity and mortality worldwide with over 650 million affected adults^1,2^, has attracted interest in preventive research of various study designs in light of its impact on the healthcare system and the economy^3,4^. However, it is challenging to encourage patients to take part in randomized trials, in part because of the perception that participation in control group may not be valuable^5^. With a median dropout rate of 24%, difficulties in recruiting, retaining and obtaining outcome data from participants are common in lifestyle randomised controlled trials (RCTs)^6–8^ and they contribute to trials being underpowered or invalid. There is a need to generate information about benefits of participation in trials to enthuse participants to engage in obesity research in a manner that robust and timely results can be produced to inform future practice and policy^9^.

A literature search demonstrated that participants of RCTs, on average, experienced better outcomes compared with those outside trials^10–15^. There is a scarcity of reviews concerning participation in lifestyle modification research^16^, and none is focused in overweight or obese participants being at risk of a chronic disease to assess benefits of clinical trials based in diet in the last decade. Descriptions of treatment and outcomes of control groups participants have received limited attention^17,18^. In obesity research it would be important to know if control groups experience any benefits inside RCTs, not only to encourage participation, but also to interpret findings of trials on effect of participation, with respect to intragroup differences in control and intervention groups. In this systematic review and meta-analysis, we aimed to determine whether participants with overweight, obesity or metabolic syndrome, allocated to control groups in lifestyle modification research experienced benefits in terms of weight loss during the course of the RCTs.

## Material and methods

We performed the systematic review after prospective registration (PROSPERO number: CRD42021233070) and reported it in accordance with relevant guidelines^19^.

### Search and selection

We conducted a comprehensive literature search without language restrictions in electronic databases (Medline via ProQuest, Scopus, Web of Science, Cochrane library and Clinicaltrials.gov) from inception to May 2021. In addition, we hand-searched reference lists of previous reviews and included articles. The search term combination was based on MeSH terms, free-text words and word variants. The inclusion criteria lifestyle intervention RCTs based on diet, with or without physical activity, and with or without behavioural support, among adults with overweight, obesity or metabolic syndrome. In crossover RCTs, control group participants were on a waiting list with standard care to receive further intervention after a wash-up period. The combination of keywords and terms included: metabolic syndrome, obesity, overweight, diet, hypocaloric diet, Mediterranean diet, physical activity, educational intervention, preventive program, diabetes mellitus, cancer, cardiovascular disease, weight loss, mortality, randomized controlled trial, lifestyle intervention, lifestyle modification, lifestyle risk reduction (Appendix 1). All citations found were exported to Endnote where duplicates were removed.

Two reviewers (ABH and PMG) carried out a search strategy independently using electronic databases and manual searches. Both of them screened all abstracts and titles. Exclusion criteria were studies conducted on children, adolescents and pregnant women; participants with established cardiovascular disease, cancer, diabetes or eating disorders; sample selection based on special conditions like familiar hypercholesterolemia o bariatric surgery, polycystic ovary syndrome, kidney disease or chronic obstructive pulmonary disease. We also excluded studies with no control group or those which did not provide outcome data for the control group. Study designs other than RCT and types of interventions other than lifestyle modification (like drug treatments or diet supplements) were excluded. Any disagreement regarding the articles’ inclusion was resolved by taking the opinion of a third researcher (NCI). We contacted authors to achieve not available full text articles. Finally, the selection of articles was based on independent review of full texts to ensure the inclusion and exclusion criteria have been fulfilled.

### Data extraction and risk of bias

The key characteristics of selected studies were extracted independently by both reviewers (ABH and PMG) after reading the full text. We used a predefined form for data extraction and, when necessary, we contacted directly the authors through ResearchGate for relevant data that were not provided in the manuscripts. Jadad scale (score range 0–5)^20^ was used to assess the methodological quality of randomization, blinding and patient withdrawals or dropouts. RCTs with a score of ≥ 3 was considered to be of high quality. We used this scale because the features assessed apply to control group, and also it has allowed us to verify the overall quality of the trials included. Given the type of lifestyle interventions used in these RCTs, double-blind was not possible. Disagreement was resolved by discussion between both reviewers or consultation with the third reviewer.

### Data synthesis and statistical analysis

We used the outcomes of the control groups reported by the authors as the mean difference in kg of body weight lost from baseline to post-participation and its standard deviations (SD). In three reviews^21–23^, which is the 13.6% of the studies, did not provide explicitly the mean difference. We calculated the weight change from the mean values reported by the authors for control group at basal and post-participation time in the RCT. We calculated the standard deviation (SD) using the confidence interval (CI) with this formula: SD = $\overline{x}$ ± *tc* (*s/*√n). Meta-analysis was deployed to comply with the recommended statistical approach, ensuring that the same metric unity (kg) was used to estimate mean difference and that the effect of the advice to control group was comparable across trials^24^, constructing forest plots with Stata v.15 software (Stata Corp., 2015, College Station, TX, USA). A random effects model was performed since each study provides information about a different effect size. We attempted to ensure that all these effect sizes are represented in the summary, and did not remove a small study by giving it a very small weight, as it would be done in a fixed-effect analysis. Heterogeneity among studies was assessed using Q test and I-squared (I^2^) statistics. We assumed that an I^2^ > 50% indicates substantial heterogeneity and I^2^ > 75% considerable heterogeneity^25,26^. In order to find out whether control group counselling was sufficiently similar across trials, we followed the criteria established by the *Cochrane Handbook for Systematic Reviews of Interventions*^27^. Subgroup meta-analysis was performed stratifying by follow-up period, type of control group protocols, and high-quality studies.

## Results

### Study selection and quality assessment

**Figure 1:**
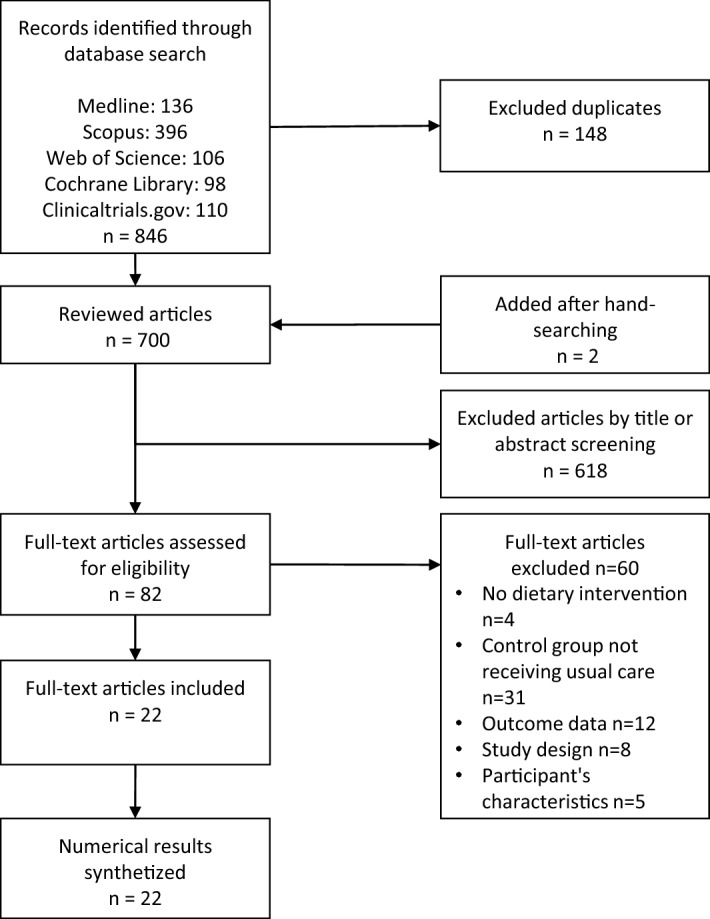
Flow chart of lifestyle randomized controlled trials’ selection.

**Figure 2:**
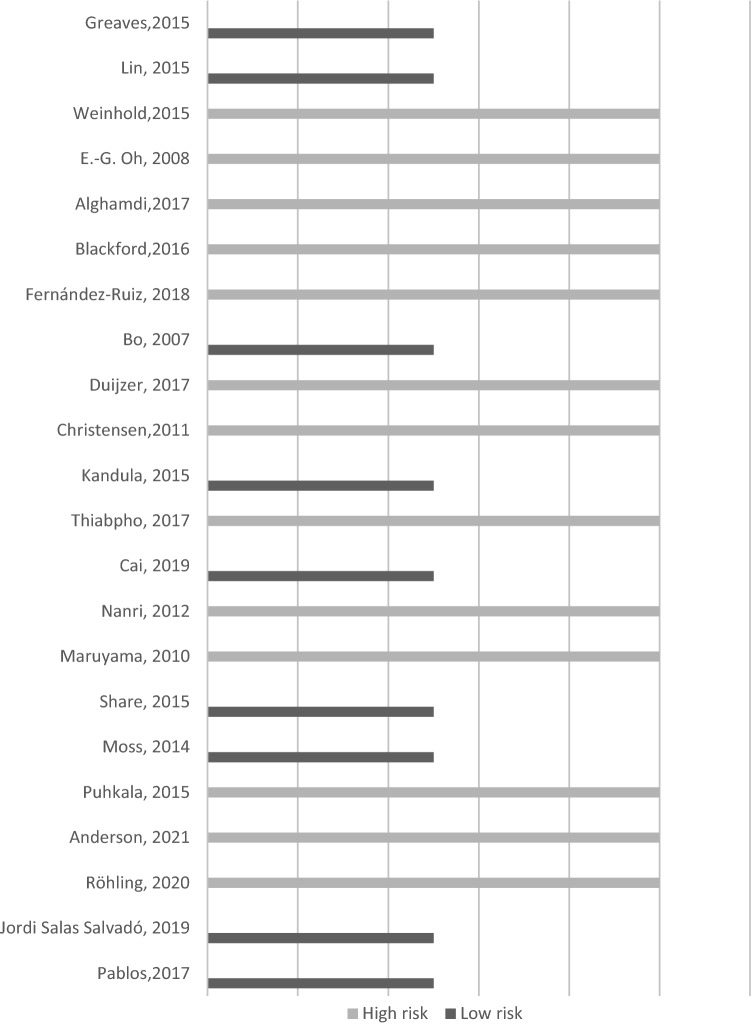
Quality assessment of the studies included in the review using Jadad scale.

A total of 846 records were identified initially. In total, 22 studies with 4032 participants were finally included (Fig. 1). The main characteristics of the studies included are summarized in Table 1. In all RCTs a lifestyle intervention was performed. The studies were conducted in several countries United Kingdom (3), United States (3), Spain (3), Japan (2), Australia (2), China (1), South Korea (1), Netherlands (1), Denmark (1), Thailand (1), Finland (1), Germany (1), Italy (1) and Saudi Arabia (1). In total, only 2 studies were published before 2010. Of the total, 9 studies (40%) were considered of high quality and 13 studies (60%) were classified as low quality (Fig. 2). Cohen's Kappa coefficient (κ) was 0.80 indicating a high inter-rater reliability between the two reviewers concerning study quality assessment.

**Table 1:** Characteristics of studies included in the review.

References	Country	Population randomized (N) and follow-up period	Patients’ characteristics	Type of intervention Lost weight objective Diet advice Physical activity advice Type of intervention Length and duration of interventions	Type of care protocols in CG	Personnel conducting intervention	Weight loss in CG Mean difference (SD) and 95% CI
Greaves^39^	UK	N = 108 12 months	Age: 40–74 BMI > 28; High CV risk	LWO: Not specified DA: Caloric and fat restriction PAA: To increase TOI: Group-based sessions L-DI: 120’ first month, followed by 90’ session at 1.5, 2, 4, 6 and 9 months	Standard care Pack of written information on CV risk	Nurse and coaches	− 2.04 (6.87) CI (− 3.96; 0.12)
Lin^40^	USA	N = 124 Outcomes at 6 months Follow up until 12 months	Age > 21 BMI > 27	LWO: Not specified DA: Reduce fat and sugar intake. Portion control PAA: Increase moving and exercise TOI: Individual L-DI: Text messages 3–4 times per day during 6 months	Standard care Initial clinical assessment, personalized weight control plan and PA recommendations. Additional educational material at 6 and 12 months	Dietitian and physician	− 0.2 (3.16) CI (− 1.4; 1.0)
Weinhold^33^	USA	N = 78 3 months	Age: 18–65 BMI = 25.0 to 50.0 Prediabetes	LWO: 7% reduction DA: Caloric and fat restriction PAA: To increase at least 150’/week TOI: Group-based L-DI: 60’/week during 3 months	Standard care Booklet with strategies for self-regulated weight loss	Dietitians	− 0.4 (0,6) CI (− 0.59; − 0.21)
Oh^22^	South Korea	N = 32 1 month	Age > 20 Rural women with MetS	LWO: Not specified DA: Caloric and saturated fat restriction PAA: Strength training, rhythmic dance, warm up, and cooldown exercises TOI: Group-based L-DI: 12 sessions, 3 times/week, 120’/session during 1 month	Standard care Educational booklet	Nurses	− 2 (10.90) CI (− 8.59; 4.59)
Alghamdi^41^	Saudi Arabia	N = 70 3 months	Age > 20 BMI ≥ 30	LWO: ≥ 5% reduction DA: Caloric and CH restriction PAA: To increase TOI: Individual sessions L-DI: 8 visits (15–20’ each) during 3 months	Standard care Printed health education materials regarding diet and PA	Nurses	− 2.8 (4.96) CI (− 3.96; − 1.64)
Blackford^44^	Australia	N = 401 6 months	Age: 50–69 Rural adults with, or at risk of MetS	LWO: Not specified DAA: Diet intervention with motivational support PAA: To increase TOI: Home-based: printed and interactive online material LDI: Self-management during 6 months	Waitlisted to receive the programme after post-test data collection	Home-based	1.1 (21.95) CI (− 2.29; 4.49)
Fernández-Ruiz^35^	Spain	N = 74 12 months of intervention, and 1-year follow-up post-intervention	Age: not defined BMI = 25.0–29.9 or BMI > 30	LWO: Not specified DAA: Modification of unhealthy dietary habits PAA: To increase: stretching exercises followed by moderate aerobic work TOI: Group-based L-DI: Monthly session (60’) for educational treatment. Four sessions (40’) of PA every week. Monthly session (6’) of cognitive behavioural therapy	Standard care	Physicians, nurses, nutritionists and psychologists	− 0.2 (12.35) CI (− 4.18; 3.78)
Bo^36^	Italy	N = 335 1 year	Age: 45–64 Adults with MetS	LWO: Not specified DA: Individually prescribed diet PAA: To increase 150’/week TOI: Individual and group-based L-DI: 5 sessions of 60’: 1 individual session and 4 grouped	Standard care	Family physicians and dietitian	1.63 (6,17) CI (0.83; 2.42)
Duijzer^34^	Netherlands	N = 316 18 months	Age: 40–70 High risk of type 2 diabetes	LWO: < 5–10% DA: Tailored dietary advice PAA: To increase at least 30’/day, 5 days/week TOI: Individual and group-based L-DI: 5 to 8 individual consultations and one group session	Standard care	General practitioners, practice nurses, dieticians and physiotherapists, sport coaches	− 0.4 (3.7) CI (− 1.06; 0.26)
Christensen^29^	Denmark	N = 144 12 months (results of the first 3 months)	Age: 18–40 (BMI > 25 or body fat % > 33) Age > 40 years (Body fat % > 34) Female health care workers	LWO: Not specified DA: Caloric restriction PAA: To increase TOI: Individual and group-based L-DI: 180’/week	Standard care Monthly two-hour oral lecture	Sport instructors	0.68 (2.37) CI (− 0.02; 1.38)
Kandula^42^	USA	N = 63 6 months	Age: not defined Participants with at least one atherosclerotic CV risk factor, including obesity	LWO: Not specified DAA: Fat and salt restriction PAA: To increase 150’/week of moderate intensity TOI: Group-based classes and individual follow-up telephone support calls L-DI: weekly group classes (60–90’) and individual telephone support during 4 months	Standard care Translated print education materials about atherosclerotic CV risk and healthy behaviours	Dieticians	− 0.2 (3,13) CI (− 1.14; 0.78)
Thiabpho^30^	Thailand	N = 60 4 months	Age: 30–50 (BMI)⩾27.5 With no non-communicable disease	LWO: Not specified DAA: Caloric restriction and balanced diet PAA: To increase a minimum of 150’/week of moderate exercise TOI: Group-based L-DI: During 4 months 12 sessions (90–120’), once a week for the first eight weeks and then every two weeks until the 16th week	Standard care	Nurses	− 0.7(1.4) CI (− 1.20; − 0.20)
Cai^38^	China	N = 480 24 months	Age: ≥ 60 BMI ≥ 28	LWO: Not specified DA: Caloric, fat and sugar restriction PA: To increase TOI: Group-based and individual based interventions L-DI: Group-based sessions (120’/week the first 12 months; 120’ monthly the following months	Standard care 2-h education sessions every 2 months	Dietitians	− 0.03 (2.51) CI (− 0.37; 0.31)
Nanri^28^	Japan	N = 107 6 months	Age: not defined Men diagnosed with MetS	LWO: Not specified DA: Dietary change behaviours PAA: To increase TOI: Individual L-DI: Session at baseline and at 3 months	Standard care Leaflet at the baseline	Nurses	− 0.3 (7.81) CI (− 2.4; 1.8)
Maruyama^31^	Japan	N = 111 4 months	Age: 30–59 Male office workers with MetS risk factors	LWO: Not specified DA: PAA: To increase TOI: Individual and group-based L-DI: Individualized assessment and collaborative goal setting (20’ and 10’ respectively) plus 2 individual counselling sessions and monthly website advice during the 4-month period	Standard care	Registered dietitian and physical trainer	− 0.80 (2.2) CI (− 1.50; − 0.10)
Share^23^	Australia	N = 43 3 months	Age: 18–30 Women with abdominal obesity [waist circumference (WC) ≥ 80 cm], and who were physically inactive	LWO: Not specified DA: Dietary change behaviours without caloric restriction PAA: To increase 2 session/week TOI: Group-based L-DI: Weekly nutrition education and group cognitive behavioural therapy (60’)	Waitlisted to receive the programme after post-test data collection	Qualified exercise scientist, dietitian and counsellor	− 3.60 (18.67) CI (− 13.20;6)
Moss^21^	UK	N = 60 Intervention 12 weeks (3 months) and follow-up until week 26. (6,5 months)	Age: 18–85 Obese patients (BMI > 30) with at least moderate OSAHS	LWO: Not specified DA: Advice based on the principles of the eat well plate PAA: To increase: supervised exercise sessions TOI: Group-based L-DI: 3 sessions/week, then 2/week during weeks 5 to 8 and then to 1/week during weeks 9 to 12	Standard care Basic written lifestyle advice, and a weight loss leaflet	Exercise physiologist	0.2 (21) CI (− 8.11; 8.51)
Puhkala^32^	Finland	N = 113 12 months of counseling + 12 months of follow up	Age: 30–62 Male truck or bus driver, waist circumference ≥ 100 cm, absence of diabetes and little PA	LWO: < 10% reduction DA: Advice based on the principles of the eat well plate PAA: To increase 30’of moderate-intensity walking TOI: Individual L-DI: during 12 months: 6 individual sessions of 60’ and 7 telephone contacts of 30’	Standard care Advice and telephone contacts	Nutritionists and physiotherapist	− 2.5 (5.9) CI (-4.02; − 0.98)
Anderson^37^	UK	N = 560 12 months	Age: 50–70 Women with excess body weight BMI > 25	LWO: < 7% reduction DA: Personalised diet advice PAA: To increase TOI: Individual L-DI: During 12 months 2 individual sessions (60’ and 45’) in the first 3 months and then 9 (15’) support calls over the following 9 months	Standard care	Nurses	− 1.2 (5.0) CI (− 1.8; − 0.6)
Röhling^45^	Germany	N = 30 1 year	Age > 18 BMI ≥ 25	LWO: Not specified DA: Low-carbohydrate nutrition and meal replacement therapy PAA: To increase TOI: Group-based L-DI: During 3 months intervention: 7 theoretical sessions and two practical modules of 90’ each, and: 4 telephone calls (20–30’each) monthly	Waitlisted to receive the programme after post-test data collection	Nutritionists, exercise scientists, biologists, physicians and psychologists	− 1.4 (4.18) CI (− 3.3; 0.6)
Jordi Salas Salvadó^43^	Spain	N = 626 12 months	Age: 55–75 Patients without CVD, overweight/obese (BMI > 27 and < 40) and with MetS	LWO: < 5–10% reduction DA: Mediterranean diet PAA: To increase TOI: Individual and group-based L-DI: During 12 months: group sessions and telephone calls once per month	Standard care Advice about Mediterranean diet monthly without specific advice for increasing PA. Group sessions and telephone calls every 6 months	Doctors, dietitians and nurses	− 0.7 (4.07) CI (− 1.1; − 0.3)
Pablos^46^	Spain	N = 97 8 months	Age: 20–70 Adults with BMI > 25, no regular PA living in a low median household income census tract	LWO: Not specified DA: Personalized diet advice PAA: To increase TOI: Individual and group-based L-DI: 8-month intervention: 3 sessions/week of PA (140–180’) and 1 session/week of nutrition or psychological support (60’)	Waitlisted to receive the programme after post-test data collection	Doctors, nutritionists, nurses, psychologists and trainers	− 0.13 (21.48) CI (− 8.46; 8.20)

*BMI* Body mass index; *CG* Control group; *CVD* Cardiovascular disease; *MetS* Metabolic syndrome; *PA* Physical activity; *OSAHS* Obstructive sleep apnoea hypopnoea syndrome; *SD* Standard deviation; *LWO* Lost weight objective; *DA* Diet advice; *PAA* Physical activity advice; *TOI* Type of intervention; *L-DI* Length and duration of interventions.

### Characteristics of control groups

In our selected studies the sample size ranged from 32 to 626 participants, aged between 18 and 70 years old (mean age 53.92 years). Each study applied different inclusion criteria concerning the BMI. The mean of BMI was 31.93 kg/m^2^ in control group at baseline, ranging from 25.6 kg/m^2^ to 39,8 kg/m^2,21,28^. Four studies included only women^22,23,29,30^ whereas three studies enrolled only men^28,31,32^. One article^21^ set obstructive sleep apnoea hypopnoea syndrome as other inclusion criteria, while another study^33^ included prediabetic patients that have overweight or obesity. In one of our studies^34^, having overweight or obesity was not an inclusion criterion, but all participants had overweight with a BMI > 30.2 in the intervention and > 29.9 in the control group, that why we decided to include it in the review.

In six studies^30,31,34–37^ control group received only standard care, while in three studies^29,32,38^ they were given extra nutritional advice. In nine studies^21,22,28,33,39–43^ they received extra material, like written information, educational booklets or leaflets. Finally, in four studies^23,44–46^ control group participants were waitlisted to receive the programme after data extraction. The length of the follow-up ranged from 1 to 24 months. We considered as time points the end-point of the intervention provided by the authors. If these data were not available, post-intervention follow-up value was considered, like in one study^47^ where outcomes were measured at 6 months, although the follow-up lasted up to 12 months. The same criterion was applied to another study^29^, where the effects of only the first three months of intervention were reported, whilst the intervention lasted up to 12 months. In two studies^32,35^ the intervention was carried out during 12 months and, afterwards, the participants were followed up for other 12 months (post-intervention). Finally, in a RCT^21^ the intervention lasted 3 months, although the follow-up was extended to 6,5 months. The lifestyle interventions were carried out by dietitians or nutritionists in three studies^33,42,47^, and in collaboration with other health professionals (e.g., nurses, physicians, psychologists, sport coaches or trainers) in the rest of the studies. All of the RCT included physical activity (*n* = 22) as part of the intervention. Control groups received the standard or usual care, or were wait-listed to receive the lifestyle program after data collection in the RCT.

### Data synthesis

**Figure 3:**
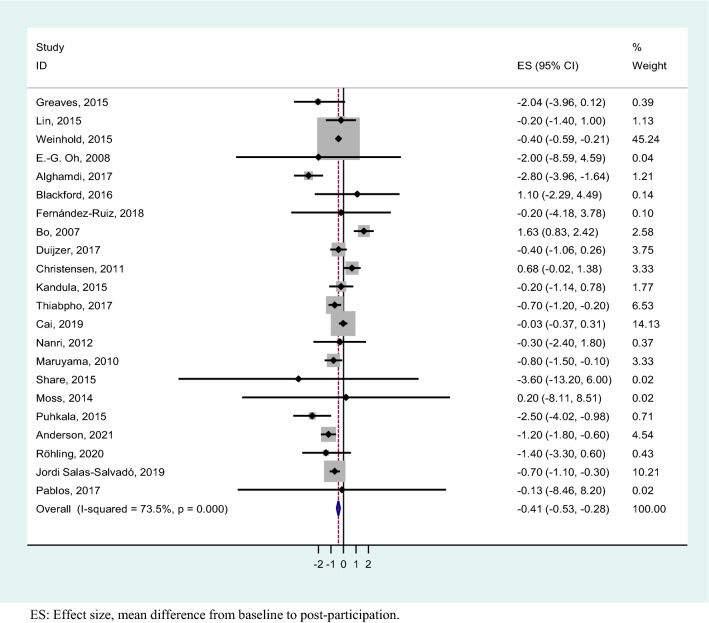
Meta-analysis of weight loss outcome in control group participants in lifestyle randomized controlled trials.

**Figure 4:**
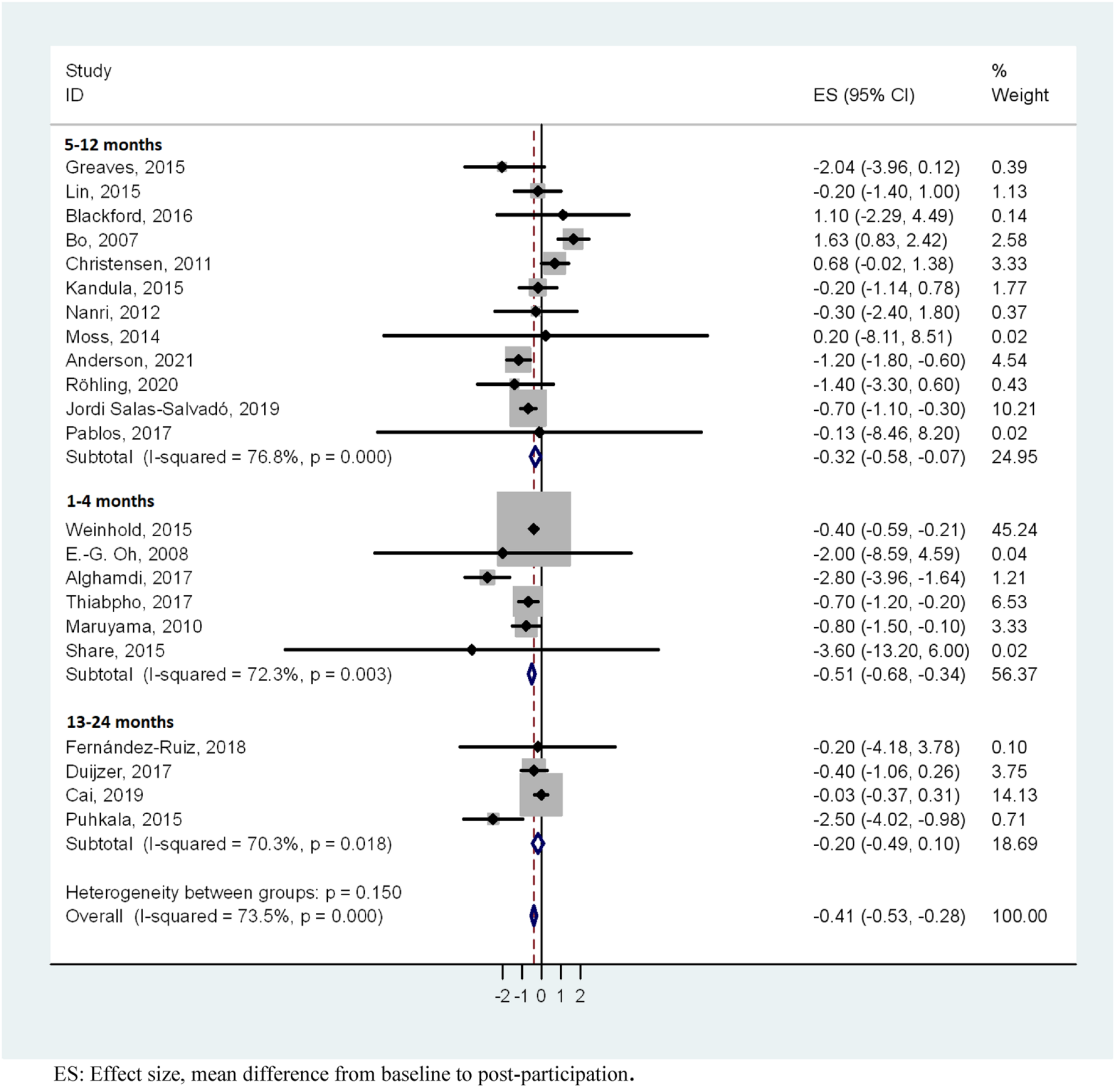
Meta-analysis of weight loss outcome in control group participants stratified by duration of follow-up in lifestyle randomized controlled trials.

**Figure 5:**
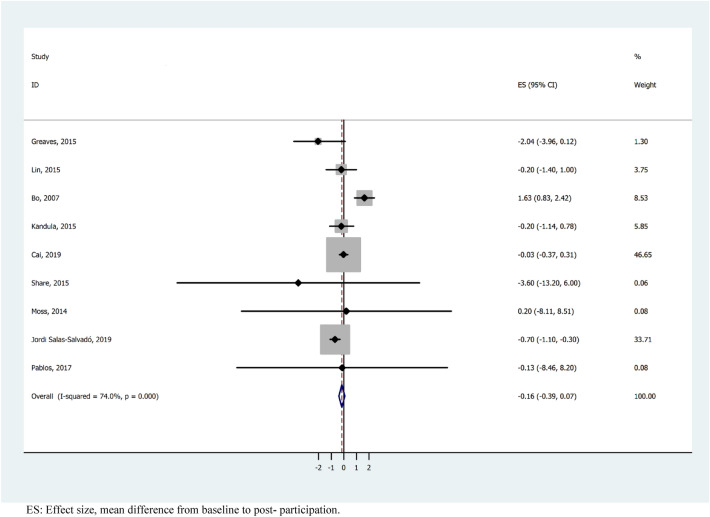
Meta-analyses of weight loss outcome in control group participants stratified by high-quality lifestyle randomized controlled trials.

**Figure 6:**
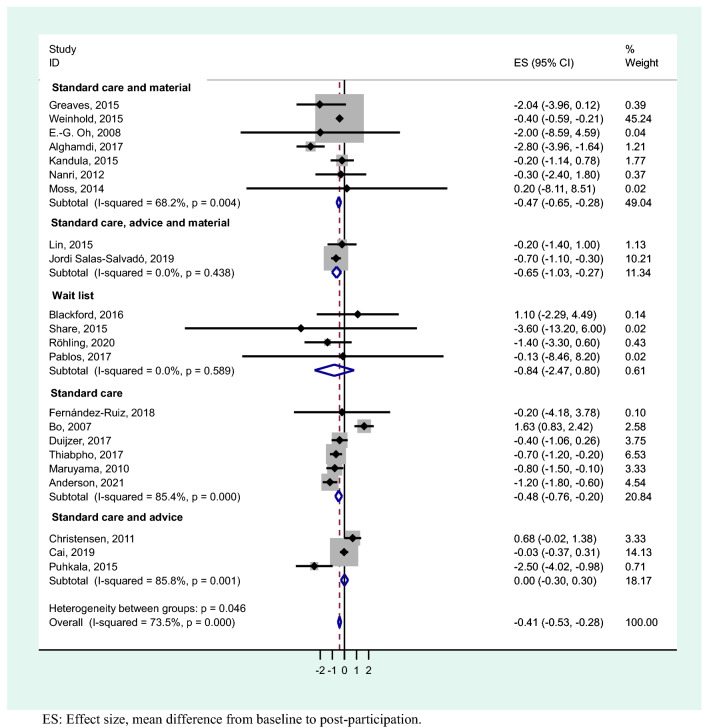
Meta-analysis of weight loss outcome stratified by type of care protocols in control group participants in lifestyle randomized controlled trials.

The results of the meta-analysis showed an overall weight loss control group mean difference of − 0.41 (95% CI − 0.53, − 0.28). These results show statistical significance with a substantial heterogeneity (*I*^2^ = 73.5%; *p* < 0.001) (Fig. 3). For studies with a follow-up period of 1–4 months, the heterogeneity was substantial (*I*^2^ = 72.3%; *p* =  < 0.003) and the mean difference was − 0.51 kg (95% CI − 0.68, − 0.34), studies with 5–12 months had a considerable heterogeneity (*I*^2^ = 76.8%; *p* =  < 0.001) and mean difference − 0.32 kg (95% CI − 0.58, − 0.07), whereas when the follow-up was > 12 months, there was a substantial heterogeneity (*I*^2^ = 70.3%; *p* =  < 0.018) and mean difference − 0.20 kg (95% CI − 0.49, 0.10) (Fig. 4). We performed a meta-analysis of high-quality studies with an overall weight loss control group mean difference of − 0.16 (95% CI − 0.39, 0.09) and a considerable heterogeneity (*I*^2^ = 74%; *p* < 0.000) (Fig. 5). As the exploration of heterogeneity leads to more meaningful, high-value conclusions, we also performed a meta-analysis comparing subgroups by type of care protocols in control group. Among studies including control group in waiting lists and combining standard care, advice and material, no heterogeneity was found (I^2^ = 0%, *p* = 0.589) and (I^2^ = 0%, *p* = 0.438), and the mean difference was − 0.84 kg (95% CI − 2.47, 0.80) and − 0.65 kg (95% CI − 1.03, − 0.27), respectively. In studies with standard care and material, the heterogeneity was substantial (I^2^ = 68,2%, *p* = 0.004) and the mean difference was − 0.47 kg (95% CI − 0.65, − 0.28). Finally, in the studies where control group participants received standard care, or standard care and advice, we found a considerable heterogeneity (I^2^ = 85.4%, *p* = 0.000) and (I^2^ = 85.8%, *p* = 0.001) with a mean difference of − 0.48 kg (95% CI − 0.76, − 0.20) and 0.00 kg (-0.30, 0.30) (Fig. 6).

## Discussion

Our meta-analysis of over four thousand participants combined showed that control groups in obesity research lost weight overall, confirming that it is safe and beneficial to participate in trials even if the allocation is not to the intervention arm.

To our knowledge, this is the first systematic review and meta-analysis focusing on control group outcomes in lifestyle intervention studies. Our findings confirm the hypothesis of health improvement of control participants, in contrast to the results on overall weight changes in a meta-regression study on behavioural weight loss interventions^16^. Our search was unrestricted, without limitations regarding language or dataset inception, to capture the highest possible number of relevant studies. There was reviewer agreement in the search, selection and quality assessment of studies adding to reliability of our work. However, our main finding was within the limitations placed by heterogeneity. This is an expected, possibly unavoidable, limitation when addressing lifestyle interventions^48^. In our review there are various possible sources of heterogeneity. Standard healthcare in control groups may vary between participants depending on the health systems in the countries where trials are carried out. We also found a diversity of approaches in handling control group engagement, e.g., providing health educational contents with a variable frequency, within the trials included, which may have different effect on weight loss^16^. With a considerable sample size, we could precisely estimate the control group weight loss. The reporting of some of the studies did not facilitate the analysis of the control group, as findings were mainly reported for intergroup differences. However, in the three mentioned articles not providing required parameters for meta-analysis, we were able to estimate them from the available data. Despite the issues arising from data reporting quality, our overall result was statistically significant.

How did the control group come to benefit? The observed benefits may be due to a trial effect, which increases adherence to care protocols^12^ and encourages interaction between patients and professionals^49^. Additionally, Hawthorne effect could improve control group outcome through modification of the behaviour of research participants just because they are observed in the course of a trial^50^. The observed fact that the control groups benefit is generally in line with the view that participating in RCTs is good for participants^10,11,16^. This finding is particularly important as the prevalent overweight and obesity rates are high. For example, in Spanish population aged 55–64 years the prevalence of overweight and obesity reaches 44% and 22% respectively^51^. As the mean age of the control group participants in Spain^35,43,46^ was 60 years, trial participation could be thought of as a strategy for weight control. The same theme is repeated for the USA, where north American studies^33,40,42^ showed a mean age of 51 years and the prevalence of overweight and obesity in the over 50 s is 70%^52^. Despite the magnitude of the effect in control group participants is not large, the fact that they experienced a weight loss inverses population trends of progressive gain during adult life^53^. According to the preventive paradox of Rose et al.^54,55^, beyond the individual benefit, this weight loss may have a high impact of the health outcomes when extended to general population, in terms of improvement of health status and reduction of burden for health systems. Health services should also consider implementing lifestyle intervention trials as part of programs for people with overweight and obesity^56^.

Lifestyle research has shown health benefits of intervention compared to control in terms of adiposity and cardiovascular risk decrease^57,58^. Our findings also show a benefit in the outcome of the control groups. Future research should examine if the benefits gained by participation in the control groups can be maintained over time as a healthy weight loss has a tendency to be gradually regained^48,59^. These benefits should be used to encourage participation in future obesity research to generate the timely evidence for practice and policy.

## Conclusions

Our systematic review showed that participation in control groups of RCTs of lifestyle interventions had a benefit in terms of weight loss in meta-analysis with heterogeneity. These results should be used to interpret the benefits observed with respect to intervention effect in trials. That control groups accrue benefits should be included in patient information sheets to encourage participation in future trials among patients with overweight or obesity.

## Supplementary Information

Supplementary Information 1.

Supplementary Information 2.

Supplementary Information 3.

Supplementary Information 4.

## Data Availability

All data generated or analysed during this study are included in this published article (Appendix 2–4).

## Abbreviations

RCTs: Randomized controlled trials
BMI: Body Mass Index
