# Author Correction: A systematic review and meta-analysis of weight loss in control group participants of lifestyle randomized trials

**DOI:** 10.1038/s41598-022-18828-y

**Published:** 2022-08-24

**Authors:** Amira Bouzalmate Hajjaj, Paloma Massó Guijarro, Khalid Saeed Khan, Aurora Bueno-Cavanillas, Naomi Cano-Ibáñez

**Affiliations:** 1 Department of Preventive Medicine and Public Health, Faculty of Medicine, University of Granada, Campus de la Salud. Avda. de la Investigación 11, 18016 Granada, Spain; 2 Preventive Medicine Unit, Universitary Hospital Virgen de Las Nieves, Granada, Spain; 3 CIBER de Epidemiología Y Salud Pública (CIBERESP-Spain), Madrid, Spain; 4 Instituto de Investigación Biosanitaria de Granada (IBS.GRANADA), Granada, Spain

Correction to: *Scientific Reports* https://doi.org/10.1038/s41598-022-15770-x, published online 18 July 2022

The original version of this Article contained errors in the Funding section.

“This research has received funding from the Ministry of Science and Innovation PI20/01532 project, and the Centro de Investigación Biomédica en Red-Epidemiología y Salud Pública (CIBERESP/CB06/02/1014).”

now reads:

“This research has received funding from the Ministry of Science and Innovation, Instituto de Salud Carlos III, FEDER co-funding from European Union (PI20/01532 project), and the Centro de Investigación Biomédica en Red-Epidemiología y Salud Pública (CIBERESP/CB06/02/1014).”

The original Article has been corrected.

