# Supplementary material for: A systematic review and meta-analysis of weight loss in control group participants of lifestyle randomized trials
Source: Sci Rep. 2022 Jul 18;12:12252. doi: 10.1038/s41598-022-15770-x (PMC9293970; doi:10.1038/s41598-022-15770-x)
Supplement: Supplementary file 1 — Supplementary Information 1. [file 41598_2022_15770_MOESM1_ESM.pdf]

Title page

A systematic review and meta-analysis of weight loss in control group participants of lifestyle randomized trials.

Running title: Control benefits in lifestyle intervention trials.

Authors and institutional affiliations:

Amira Bouzalmate-Hajjaj[1], Paloma Massó Guijarro[1,2,4], Khalid Saeed Khan[1,3], Aurora Bueno-Cavanillas[1,3,4], Naomi Cano-Ibáñez[1,3,4]

1.Department of Preventive Medicine and Public Health, Faculty of Medicine, University of Granada, Granada, Spain,

2.Preventive Medicine Unit. Universitary Hospital Virgen de las Nieves, Granada, Spain.

3.CIBER de Epidemiología y Salud Pública (CIBERESP-Spain),

4.Instituto de Investigación Biosanitaria de Granada (IBS.GRANADA)

ORCID authors:

Amira Bouzalmate Hajjaj: none, Paloma Massó Guijarro: 0000-0002-3519-9254, Khalid Saeed Khan: 0000-0001-5084-7312, Aurora Bueno-Cavanillas: 0000-0002-0649-3016, Naomi Cano-Ibáñez: 0000-0002-3640-5486

Acknowledgements:

The first author would like to acknowledge support by the CIBER Epidemiología y Salud Pública (CIBERESP/ CB06/02/1014). Professor Khan is a Distinguished Investigator at the University of Granada funded by the Beatriz Galindo (senior modality) program of the Spanish Ministry of Education.

Correspondence: Paloma Massó Guijarro.

Postal address: Department of Preventive Medicine and Public Health. Faculty of Medicine. University of Granada. Campus de la Salud. Avda. de la Investigación 11. 18016 Granada, Spain.

Email address: pmasso@ugr.es.

Funding

The research leading to these results has received funding from the Centro de Investigación Biomédica en Red-Epidemiología y Salud Pública (CIBERESP/CB06/02/1014).

Conflict of interest

The authors declare that they have no conflict of interest.

Word-character count of the complete text: 5021.

Appendix 1. Database search strings in the systematic review of lifestyle randomized controlled trials.

Database	Search string
Medline	("metabolic syndrome" OR obesity OR overweight) AND ((diet OR "hypocaloric diet" OR "restriction diet therapy" OR "mediterranean diet") AND ("physical activity" OR "educational intervention" OR "preventive program")) AND ("diabetes mellitus" OR cancer OR "cardiovascular disease" OR "weight loss" OR mortality) AND ti("randomized controlled trial") NOT (treatment OR "treatment effectiveness" OR "treatment efficacy" OR "drug treatment" OR "diet supplements" OR "pharmacology" OR "cohort studies" OR "gestational diabetes" OR "postpartum diabetes" OR "participant's perspective" OR "qualitative study" OR supplementation) NOT ti(design OR protocol OR review OR "systematic review" OR "meta-analysis" OR baseline OR rationale) AND ("lifestyle intervention" OR "lifestyle modification" OR "Lifestyle Risk Reduction")
Scopus	ALL ("metabolic syndrome") OR ALL (obesity) OR ALL (overweight) AND ((ALL (diet) OR ALL ("hypocaloric diet") OR ALL ("restriction diet therapy") OR ALL ("mediterranean diet")) AND ((ALL ("physical activity") OR ALL ("educational intervention") OR ALL ("preventive program")) AND ((ALL("diabetes mellitus") OR ALL (cancer) OR ALL ("cardiovascular disease") OR ALL ("weight loss") OR ALL (mortality)) AND (TITLE ("randomized controlled trial") AND NOT ((TITLE (design) OR TITLE (protocol) OR TITLE (review) OR TITLE ("systematic review") OR TITLE ("meta-analysis") OR TITLE (baseline) OR TITLE (rationale)) AND NOT ((TITLE-ABS-KEY (treatment) OR TITLE-ABS- KEY ("treatment effectiveness") OR TITLE-ABS-KEY ("treatment efficacy") OR TITLE-ABS-KEY ("drug treatment") OR TITLE-ABS-KEY ("diet supplements") OR TITLE-ABS-KEY ("pharmacology") OR TITLE-ABS-KEY ("cohort studies") OR TITLE-ABS-KEY ("gestational diabetes") OR TITLE-ABS-KEY (("postpartum diabetes") OR TITLE- ABS-KEY ("participant's AND perspective") OR TITLE-ABS-KEY ("qualitative study") OR TITLE-ABS-KEY (supplementation)) AND ((ALL ("lifestyle intervention") OR ALL ("lifestyle modification") OR ALL ("Lifestyle Risk Reduction"))
Web of Science	ALL=("metabolic syndrome" OR obesity OR overweight) AND ALL=((diet OR "hypocaloric diet" OR "restriction diet therapy" OR "mediterranean diet") OR ("physical activity" OR "educational intervention" OR "preventive program")) AND ALL=("diabetes mellitus" OR cancer OR "cardiovascular disease" OR "weight loss" OR mortality) AND ALL=("randomized controlled trial") AND ALL=("lifestyle intervention" OR "lifestyle modification" OR "Lifestyle Risk Reduction") NOT ALL=(treatment OR "treatment effectiveness" OR "treatment efficacy" OR "drug treatment" OR "diet supplements" OR "pharmacology" OR "cohort studies" OR "gestational diabetes" OR "postpartum diabetes" OR "participant's perspective" OR "qualitative study" OR supplementation) NOT TI=(design OR protocol OR review OR "systematic review" OR "meta-analysis" OR baseline OR rationale)

Cochrane Library	(("metabolic syndrome" OR obesity OR overweight) AND (diet OR "hypocaloric nutrition" OR "restricting oral intake" OR "restricted food pattern") AND ("physical activity" OR "preventative intervention" OR "Primary prevention")):ti,ab,kw AND (("Type 2 diabetes mellitus" OR cancer OR "cardiovascular event" OR "cardiovascular disorder" OR "weight loss" OR mortality) AND ("lifestyle intervention" OR "lifestyle modification" OR "Lifestyle Risk Reduction")):ti,ab,kw AND (("randomized controlled trial"))
