# Supplementary material for: A systematic review and meta-analysis of weight loss in control group participants of lifestyle randomized trials
Source: Sci Rep. 2022 Jul 18;12:12252. doi: 10.1038/s41598-022-15770-x (PMC9293970; doi:10.1038/s41598-022-15770-x)
Supplement: Supplementary file 4 — Supplementary Information 4. [file 41598_2022_15770_MOESM4_ESM.pdf]

Title page

A systematic review and meta-analysis of weight loss in control group participants of lifestyle randomized trials.

Running title: Control benefits in lifestyle intervention trials.

Authors and institutional affiliations:

Amira Bouzalmate-Hajjaj[1], Paloma Massó Guijarro[1,2,4], Khalid Saeed Khan[1,3], Aurora Bueno-Cavanillas[1,3,4], Naomi Cano-Ibáñez[1,3,4]

1.Department of Preventive Medicine and Public Health, Faculty of Medicine, University of Granada, Granada, Spain,

2.Preventive Medicine Unit. Universitary Hospital Virgen de las Nieves, Granada, Spain.

3.CIBER de Epidemiología y Salud Pública (CIBERESP-Spain),

4.Instituto de Investigación Biosanitaria de Granada (IBS.GRANADA)

ORCID authors:

Amira Bouzalmate Hajjaj: none, Paloma Massó Guijarro: 0000-0002-3519-9254, Khalid Saeed Khan: 0000-0001-5084-7312, Aurora Bueno-Cavanillas: 0000-0002-0649-3016, Naomi Cano-Ibáñez: 0000-0002-3640-5486

Acknowledgements:

The first author would like to acknowledge support by the CIBER Epidemiología y Salud Pública (CIBERESP/ CB06/02/1014). Professor Khan is a Distinguished Investigator at the University of Granada funded by the Beatriz Galindo (senior modality) program of the Spanish Ministry of Education.

Correspondence: Paloma Massó Guijarro.

Postal address: Department of Preventive Medicine and Public Health. Faculty of Medicine. University of Granada. Campus de la Salud. Avda. de la Investigación 11. 18016 Granada, Spain**.**

Email address: pmasso@ugr.es.

Funding

The research leading to these results has received funding from the Centro de Investigación Biomédica en Red-Epidemiología y Salud Pública (CIBERESP/CB06/02/1014).

Conflict of interest

The authors declare that they have no conflict of interest.

Word-character count of the complete text: 5021.

Appendix 4. Stata analysis commands.

Meta-analysis of weight loss outcome in control group participants in lifestyle randomized controlled trials (Figure 3):

metan meandifference1 lcl1 ucl1, label (namevar= firstauthoryear) xlabel(-2,-1,1,2)

Meta-analysis of weight loss outcome in control group participants stratified by duration of follow-up in lifestyle randomized controlled trials (Figure 4):

metan meandifference1 lcl1 ucl1, label (namevar= firstauthoryear) by (followup_months) xlabel(-2,-1,1,2)

Meta-analyses of weight loss outcome in control group participants stratified by high-quality lifestyle randomized controlled trials (Figure 5):

metan meandifference1 lcl1 ucl1, label (namevar= firstauthoryear) xlabel(-2,-1,1,2)

Meta-analysis of weight loss outcome stratified by type of care protocols in control group participants in lifestyle randomized controlled trials (Figure 6):

metan meandifference1 lcl1 ucl1, label (namevar= firstauthoryear) by (controlgroup) xlabel(-2,-1,1,2)
